# Development of Solution-Processable, Optically Transparent Polyimides with Ultra-Low Linear Coefficients of Thermal Expansion

**DOI:** 10.3390/polym9100520

**Published:** 2017-10-18

**Authors:** Masatoshi Hasegawa

**Affiliations:** Department of Chemistry, Faculty of Science, Toho University, 2-2-1 Miyama, Funabashi, Chiba 274-8510, Japan; mhasegaw@chem.sci.toho-u.ac.jp

**Keywords:** polyimides, optical transparency, low linear coefficients of thermal expansion (CTE), toughness, solution-processability, plastic substrates in image display devices

## Abstract

This paper reviews the development of new high-temperature polymeric materials applicable to plastic substrates in image display devices with a focus on our previous results. Novel solution-processable colorless polyimides (PIs) with ultra-low linear coefficients of thermal expansion (CTE) are proposed in this paper. First, the principles of the coloration of PI films are briefly discussed, including the influence of the processing conditions on the film coloration, as well as the chemical and physical factors dominating the low CTE characteristics of the resultant PI films to clarify the challenges in simultaneously achieving excellent optical transparency, a very high *T*_g_, a very low CTE, and excellent film toughness. A possible approach of achieving these target properties is to use semi-cycloaliphatic PI systems consisting of linear chain structures. However, semi-cycloaliphatic PIs obtained using cycloaliphatic diamines suffer various problems during precursor polymerization, cyclodehydration (imidization), and film preparation. In particular, when using *trans*-1,4-cyclohexanediamine (*t*-CHDA) as the cycloaliphatic diamine, a serious problem emerges: salt formation in the initial stages of the precursor polymerization, which terminates the polymerization in some cases or significantly extends the reaction period. The system derived from 3,3′,4,4′-biphenyltetracarboxylic dianhydride (s-BPDA) and *t*-CHDA can be polymerized by a controlled heating method and leads to a PI film with relatively good properties, i.e., excellent light transmittance at 400 nm (*T*_400_ = ~80%), a high *T*_g_ (>300 °C), and a very low CTE (10 ppm·K^−1^). However, this PI film is somewhat brittle (the maximum elongation at break, *ε*_b max_ is about 10%). On the other hand, the combination of cycloaliphatic tetracarboxylic dianhydrides and aromatic diamines does not result in salt formation. The steric structures of cycloaliphatic tetracarboxylic dianhydrides significantly influence the polymerizability with aromatic diamines and the CTE values of the resultant PI films. For three isomers of hydrogenated pyromellitic dianhydride, the steric structure effect on the polymerizability and the properties of the PI films is discussed. 1,2,3,4-Cyclobutanetetracarboxylic dianhydride (CBDA) is a very unusual cycloaliphatic tetracarboxylic dianhydride that is suitable for reducing the CTE. For example, the PI system derived from CBDA and 2,2′-bis(trifluoromethyl)benzidine (TFMB) yields a colorless PI film with a relatively low CTE (21 ppm·K^−1^). However, this PI is insoluble in common organic solvents, which means that it is neither solution-processable nor compatible with the chemical imidization process; furthermore, the film is somewhat brittle (*ε*_b_ < 10%). In addition, the effect of the film preparation route on the film properties is shown to be significant. Films prepared via chemical imidization always have higher optical transparency and lower CTE values than those prepared via the conventional two-step process (i.e., precursor casting and successive thermal imidization). These results suggest that compatibility with the chemical imidization process is the key for achieving our goal. To dramatically improve the solubility in the CBDA-based PI systems, a novel amide-containing aromatic diamine (AB-TFMB), which possesses the structural features of TFMB and 4,4′-diaminobenzanilide (DABA), is proposed. The CBDA(70);6FDA(30)/AB-TFMB copolymer has an ultra-low CTE (7.3 ppm·K^−1^), excellent optical transparency (*T*_400_ = 80.6%, yellowness index (YI) = 2.5, and haze = 1.5%), a very high *T*_g_ (329 °C), sufficient ductility (*ε*_b max_ > 30%), and good solution-processability. Therefore, this copolymer is a promising candidate for use as a novel coating-type plastic substrate material. This paper also discusses how the target properties can be achieved without the help of cycloaliphatic monomers. Thus, elaborate molecular design allows the preparation of highly transparent and low-CTE aromatic poly(amide imide) and poly(ester imide) systems.

## 1. Introduction

Heat-resistant polymers, which are representative high-performance polymers, have been used as dielectric layer materials in flexible printed circuits. They have also supported the recent technological development of flexible optoelectronics [1,2]. For example, organic light-emitting diode (OLED) display devices are fabricated by forming various micro-components (e.g., thin-film transistors) through ink-jet and screen-printing methods on heat-resistant polymer substrates. Recent demand for these materials has been directed toward optical and optoelectronic applications. Optically transparent (colorless) heat-resistant polymers are key materials for plastic substrates in image display devices, liquid crystal alignment layers, color filters, optical compensation films, optical fibers, light-guiding plates, and optical lenses. In addition, plastic substrates (<50 μm thick) alternative to the current inorganic glass substrates (400–700 μm thick) are urgently required with the aim of lightening, thinning, and toughening of image display devices. However, there are no reliable plastic substrate materials available yet because of the great difficulty in simultaneously achieving excellent optical transparency, heat resistance, dimensional stability, film ductility, and film-forming process compatibility. This paper reviews the research and development of new high-temperature polymeric materials suitable for use as plastic substrates in display devices with a focus on our previous results.

Unfortunately, commercially available colorless super-engineering plastics are not applicable because of their insufficient physical (short-term) heat resistance, which is represented by the glass transition temperature (*T*_g_); for example, even poly(ether sulfone) (PES), which has the highest *T*_g_ (225 °C [3]) of these materials, is not sufficient. Although the *T*_g_s of plastic substrate materials should be as high as possible (a realistic target is *T*_g_ > 350 °C), a more desirable value is *T*_g_ > 400 °C or the absence of prominent softening behavior up to 400 °C (no detection of distinct *T*_g_ by dynamic mechanical analysis).

In addition to the heat resistance, the dimensional stability to temperature change has become desirable in recent years. Plastic substrates with poor dimensional stability suffer significant thermal expansion/contraction during the heating–cooling cycles in the device fabrication processes, which causes serious problems, e.g., misalignment and adhesion failure of various micro-components, laminate warpage, and transparent electrode breakdown. A direct strategy for enhancing the dimensional stability to temperature change is to reduce the linear coefficients of thermal expansion (CTE) of the substrate materials in the glassy temperature regions (*T* < *T*_g_) along the *X*–*Y* (film plane) direction (desirably, a CTE of less than 10 ppm·K^−1^, although ideally zero CTE). In this regard, PES is insufficient as the plastic substrate because it has a high CTE value of 60 ppm·K^−1^, as often seen in common polymers (50–120 ppm·K^−1^) [4].

In contrast, aromatic polyimides (PIs) have satisfactory heat resistance, and it is not difficult to obtain very high *T*_g_s (>350 °C) by enhancing their chain rigidity. Therefore, aromatic PIs have been used as electrical insulation materials in a variety of electronic devices [5,6,7,8,9,10]. Some of the commercially available PI films are known to have not only excellent heat resistance but also low-CTE characteristics (e.g., Uplilex-S^®^, Kapton-EN^®^, and Apical-NPI^®^). However, aromatic PI films have a significant problem: they are intensely colored (e.g., yellowness index, YI = 46.0 for a 15 μm thick Upilex-S^®^ film). This color arises from charge-transfer (CT) interactions [11]. The film coloration often prevents optical applications. Therefore, except for applications as the plastic substrates in top-emission-type OLED display devices [12], the current aromatic PI films are unsuitable for use as plastic substrate materials. Special attention has been paid to eliminating the coloration of PI films for optoelectronic [13] and space applications [14]. Furthermore, it is difficult to achieve excellent optical transparency and low-CTE characteristics simultaneously. However, as a promising solution to this problem, nano-composites containing inorganic nano-fillers or nano-fibers have been investigated. However, we have so far concentrated on the development of practically useful polymeric materials with the target properties without the assistance of any fillers. In this review paper, molecular design strategies for the development of new plastic substrate materials with excellent combined properties (i.e., excellent transparency, high *T*_g_, very low CTE, good film ductility, and sufficient solution-processability) are proposed. This paper also describes the important issues encountering in the PI film preparation processes and the resultant film properties.

## 2. Optically Transparent Polyimides

### 2.1. Factors Influencing the Transparency of PI Films

The degree of coloration of PI films is evaluated by the total light transmittance (*T*_tot_), yellowness index (YI, ASTM E 313), and haze (turbidity). More conveniently, the light transmittance at a wavelength of 400 nm (*T*_400_), which can be measured by an ultraviolet-visible (UV-vis) spectrometer, is also useful in estimating the degree of film coloration. For the 20–30 μm thick films, a provisional target of the optical properties is established: YI < 3 and haze < 1.0%. When a film has a high *T*_400_ (>80%), it usually has a very low YI value (<3). However, this condition (*T*_400_ > 80%) is not always indispensable for obtaining a very low YI value (<3) because, as mentioned later, there exist almost colorless PI films that have not very high *T*_400_ values (40–50%) [15]. In particular, note that the light transmittance at longer wavelengths such as 450 or 500 nm is a less useful parameter for evaluating the film coloration because the films are not always colorless even if the *T*_500_ value is very high (e.g., *T*_500_ > 80%).

The extent of coloration and turbidity of PI films are affected by the following factors.

(a) Chemical factors
  - CT interactions and electronic conjugation originating from the PI chain structures
  - Partial decomposition of terminal amino groups, aliphatic units in the main chains and the side groups
(b) Physical factors
  - Chain aggregation influenced by imidization methods (film preparation routes) and heating programs for film formation
(c) Processing factors
  - Temperatures, atmosphere, and type of solvent(s) used for film preparation
  - Unknown colored impurities originally contained in the monomers, particularly aromatic diamines.

The chemical structure of the PIs is a primary factor affecting the degree of film coloration, as described later. However, the technical factors such as processing conditions should also be examined to ensure excellent optical transparency of the films.

### 2.2. Influences of Processing Conditions

The optical transparency of PI films is often affected by unexpected factors that are not related to the backbone structure of the PI. For example, when PIs have insufficient molecular weights (*M*_w_), they often contain a non-negligible amount of unstable amino groups at the chain ends. Thermal and light-induced decomposition of the terminal amino groups often produces unknown colored products in the films. However, end-capping treatment using mono-functional reagents (e.g., phthalic anhydride and acetic anhydride (Ac_2_O)) effectively prevents this problem [16].

In the PIs containing aliphatic units, the heating conditions and atmosphere are also important. Heat treatment in air often causes significant coloration. Even in inert gases or a vacuum, in PI systems including cycloaliphatic units, the films often become abruptly colored on heating above 330–350 °C. Some aromatic diamines [e.g., 4,4′-diaminobenzanilide (DABA) as a typical case, and sometimes *p*-phenylenediamine (*p*-PDA) as well] from commercial sources are often intensively colored because of the presence of trace amounts of unknown colored impurities. The use of these diamines often causes significant coloration of the resultant PI films. Higher chemical purities do not always correspond to lower contents of colored impurities when compared to the same diamines from different sources. Sublimation under a reduced pressure is more effective for the decoloration of aromatic diamines [particularly for *p*-PDA and 4,4′-oxydianiline (4,4′-ODA)] than the recrystallization method using suitable solvents. In particular, the decoloration of DABA as a pre-treatment is very difficult, even by recrystallization in the presence of activated carbon and a small amount of water.

**Figure 1:**
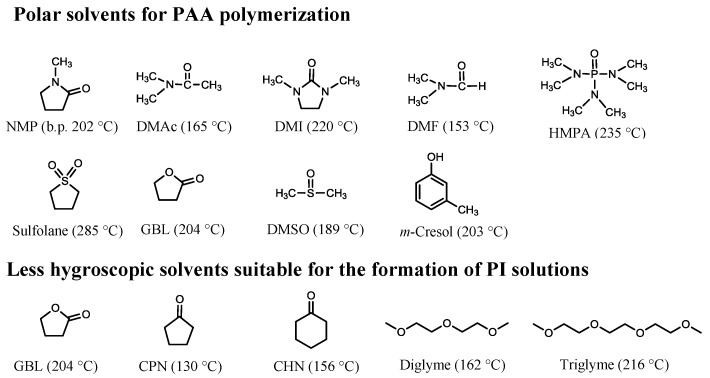
Polar solvents used for polymerization of PAAs and less hygroscopic solvents used for re-dissolution of chemically imidized powder samples. NMP = *N*-methyl-2-pyrrolidone, DMAc = *N*,*N*-dimethylacetamide, DMI = 1,3-dimethyl-2-imidazolidinone, DMF = *N*,*N*-dimethylformamide, HMPA = hexamethylphosphoramide, GBL = *γ*-butyrolactone, CPN = cyclopentanone, CHN = cyclohexanone.

The choice of solvents is also important. Figure 1 shows the solvents that are often used in the polymerization of PI precursors [poly(amic acid) (PAAs)] and for the formation of PI solutions by dissolving the chemically imidized powder samples. The abbreviations of the solvents are shown in the figure caption. *N*-Methyl-2-pyrrolidone (NMP) is usually used for PAA polymerization because of its higher dissolution ability and lower toxicity than other polar solvents. However, amide solvents such as NMP tend to cause appreciable film coloration, probably owing to the generation of unknown colored products by oxidation at elevated temperatures. NMP is also disadvantageous compared to DMAc regarding film coloration. When PI films are prepared via the conventional two-step process [precursor casting + thermal imidization (cyclodehydration)] under vacuum, empirically speaking, the degree of coloration in the resultant PI films, which are originally colorless, decreases in the following order: HMPA >> NMP > DMAc ≥ GBL and triglyme. This trend is probably related to their oxidative stability and the difficulty in evaporation from the film during thermal imidization (i.e., the period that the solvent remains in the films). The latter is dominated by the combined effect of their boiling points and the strength of the PAA–solvent and PI–solvent interactions. Based on our experience, HMPA should not be used. For example, 1,2,3,4-cyclobutanetetracarboxylic dianhydride (CBDA) and *trans*-1,4-cyclohexanediamine (*t*-CHDA) could be polymerized only in a mixed solvent of HMPA and DMAc [17]. However, when the precursor cast film including the solvents was thermally imidized on a substrate under vacuum, unexpectedly, a black-colored film was formed, probably owing to the formation of a strongly colored unknown product by partial thermal decomposition of HMPA. Therefore, a solvent extraction process is indispensable for avoiding such prominent coloration before thermal imidization [17].

GBL is the most favorable solvent for our purposes because of the combination of its moderate dissolution ability, ease of evaporation from the films despite its high boiling point, and oxidative stability. In addition, the lower hygroscopic property of GBL is advantageous because, in contrast to amide solvents such as NMP and DMAc, a clouding phenomenon of the coatings by moisture absorption, which can cause the hazing of the resultant PI films, is avoided during the casting process. This superiority is also valuable in simplifying the coating facility; i.e., the coating process can be carried out without any dry chambers.

**Figure 2:**
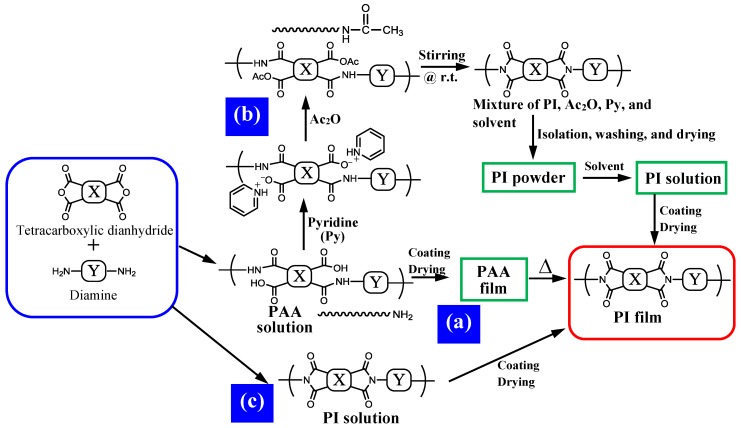
The schemes of PAA polymerization, imidization, and film preparation via different pathways: (a) two-step method (thermal imidization of PAA cast films), (b) chemical imidization method, and (c) one-pot method.

The degree of coloration of PI films also often strongly depends on the imidization method (the routes of PI film preparation). There are three pathways for PI film preparation, as shown in Figure 2. In insoluble PI systems (the systems using cycloaliphatic diamines mostly belong to this category), the film preparation method is confined to the conventional two-step process via thermal imidization of the PAA cast films (Figure 2(a)). On the other hand, highly soluble PI systems are compatible with not only the two-step process but also the chemical imidization method using a dehydrating reagent (Figure 2(b)) and the one-pot method (Figure 2(c)) by refluxing the monomer solutions or the PAA solutions. The one-pot method is the simplest process among the three routes, representing a “short-cut” process for obtaining PI films. However, it is necessary to establish the polymerization conditions (monomer contents, solvents, catalysts, azeotropic reagents, heating rates, reaction temperatures and time) carefully to obtain homogeneous solutions of PIs with sufficiently high *M*_w_s [18,19]. As shown in Figure 2(b), the film preparation process via chemical imidization is somewhat complicated, compared to the one-pot process. However, the former is more suitable for small-scale evaluation using novel homemade monomers than the latter because the chemical imidization can be very simply carried out by magnetic stirring at room temperature.

Chemical imidization is carried out by adding an excess quantity of the dehydrating reagent (Ac_2_O/pyridine) very slowly into PAA solutions and stirring at room temperature for 12–24 h. However, this process can only be applied to highly soluble PI systems because, otherwise, the reaction mixtures suffer gelation or precipitation during the reaction, whereby imidization is not completed. The chemically imidized PIs are isolated as a fibrous powder by dropping the obtained homogeneous reaction mixture very slowly into a large quantity of poor solvent (e.g., methanol, water, or a mixture), and subsequently, washing and drying. The excess dehydrating reagent should be completely removed in this process because, in some cases, the residual dehydrating reagent acts as a trigger for the coloration of the resultant PI films. The completion of chemical imidization is confirmed by the disappearance of the NHCO (*δ* ~ 10 ppm) and COOH (*δ* ~ 13 ppm) proton signals originating from the PAAs in the ^1^H-NMR spectra. The chemically imidized powder samples are usually highly soluble in common solvents and form stable PI solutions at room temperature with high solid contents sufficient for the subsequent solution casting process.

**Figure 3:**
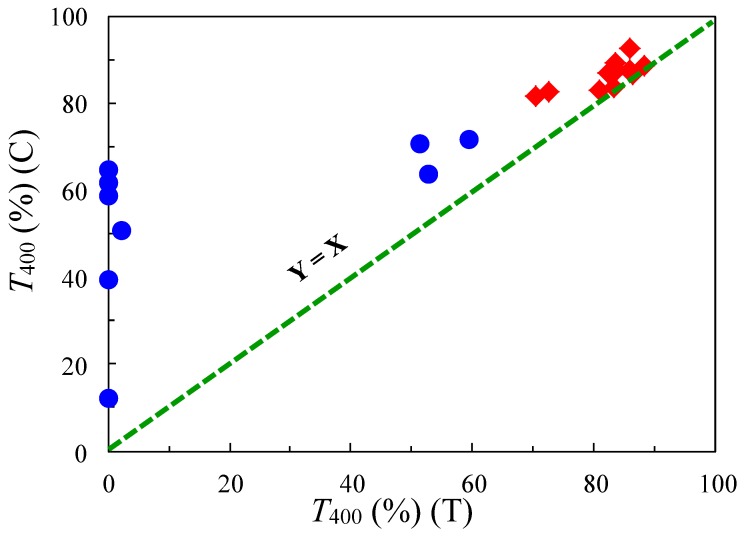
Effect of imidization method [chemical imidization (C) and thermal imidization (T)] on light transmittance at 400 nm (*T*_400_). (●) PEsIs derived from substituted TA-HQ and TFMB and (♦) H″-PMDA-based PIs. The dotted line denotes the relationship: *Y* = *X*. Reproduced with permission from Elsevier [21].

The compatibility with the chemical imidization process brings another positive effect concerning the PI film transparency. Figure 3 shows a comparison of the optical transparency (*T*_400_) between PI films with the same structures prepared by different imidization methods: chemical imidization (C, ordinate) and thermal imidization (T, abscissa) for cycloaliphatic poly(ester imide)s (PEsI) and semi-cycloaliphatic PIs that we have studied so far [20,21]. The comparison is confined to highly soluble PI systems where no gelation occurs during chemical imidization. The results show that the plots are mostly positioned in the region above the *Y* = *X* line, indicating that the film preparation method via chemical imidization is superior to the conventional two-step process via thermal imidization in terms of the optical transparency of the PI films. This clear trend probably results from the fact that the unstable terminal amino group can be end-capped by Ac_2_O during chemical imidization, as shown in Figure 2(b). Another advantage in this film preparation route is that the PI films can be formed by coating and drying at much lower temperatures (≤250 °C) than in the thermal imidization process (≥300–350 °C).

### 2.3. Influence of the Chain Structure on the PI Film Transparency

**Figure 4:**
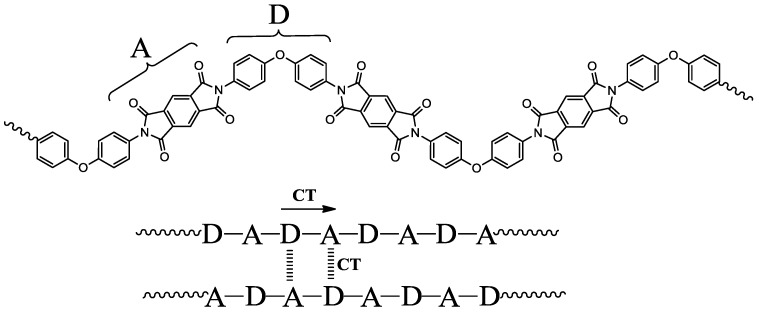
Chain sequence of wholly aromatic PIs (PMDA/4,4′-ODA system is shown here as an example) and intra- and intermolecular CT interactions. D and A represent electron-donating and electron-accepting units, respectively.

Wholly aromatic PI main chains are regarded as having an alternatingly connected sequence consisting of an electron donor (*N*-aromatic unit) and an electron acceptor (diimide unit), as schematically depicted in Figure 4. The intra- and intermolecular donor–acceptor CT interactions cause a new absorption band, which is different from that of each component, in the visible wavelength range. This is responsible for the coloration of aromatic PI films [11]. Incidentally, in the precursors of aromatic PIs (i.e., PAAs), there are weak CT interactions [22]. The coloration behavior in aromatic PIs also complies with Mulliken’s CT theory [23], which has been developed for intermolecular CT complexes in solutions between low-*M*_w_ electron donors and acceptors. This theory is expressed by the relationship:

*hν*_CT_ (∝ *λ*_CT_^−1^) = *I*_P_ − *E*_A_ + *C* (1)

where *hν*_CT_ is the energy of CT absorption transition, which is proportional to the reciprocal (peak) wavelength of the CT absorption band (*λ*_CT_^−1^), *I*_P_ is the ionization potential of the electron donor, *E*_A_ is the electron affinity of the electron acceptor, and *C* is a constant. The CT fluorescence behavior also obeys this theory. Indeed, for a series of aromatic PIs derived from a fixed diamine and different tetracarboxylic dianhydrides, a good linear relationship is observed between the reciprocal peak wavelengths of the CT fluorescence bands and the *E*_A_ values of the tetracarboxylic dianhydrides used, which are close to those of the diimide units in the PI chains [24]. According to Equation (1), the use of the diamines with higher *I*_P_ values and the tetracarboxylic dianhydrides with lower *E*_A_ values increases the energies of the CT absorption transitions, corresponding to a blue-shift of the CT band. Consequently, the coloration of PI films is reduced.

**Figure 5:**
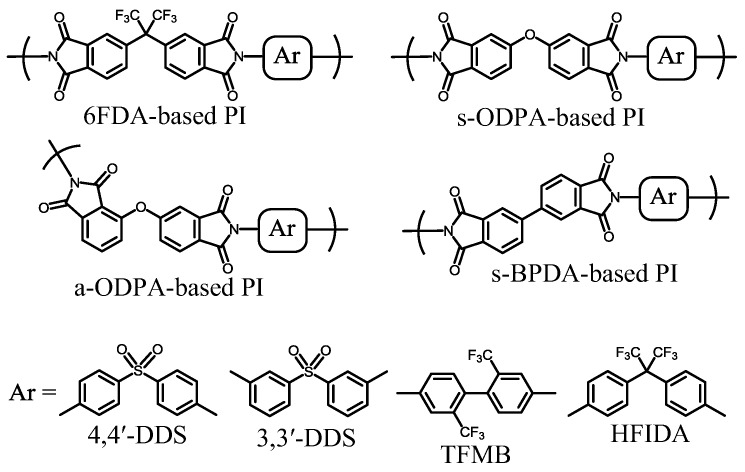
The structures of aromatic PI systems leading to non- or slightly colored films. 6FDA = 4,4′-(hexafluoroisopropylidene)diphthalic anhydride, s-ODPA = 4,4′-oxydiphthalic anhydride, a-ODPA = 3,4′-oxydiphthalic anhydride, s-BPDA = 3,3′,4,4′-biphenyltetracarboxylic dianhydride, 4,4′-DDS = 4,4′-diaminodiphenylsulfone, 3,3′-DDS = 3,3′-diaminodiphenylsulfone, TFMB = 2,2′-bis(trifluoromethyl)benzidine, HFIDA = 4,4′-(hexafluoroisopropylidene)dianiline.

Thus, to reduce film coloration, it is effective to introduce electron-withdrawing groups to aromatic diamines, and by contrast, electron-donating groups to aromatic tetracarboxylic dianhydrides. Some of the practically useful aromatic PIs with no or low coloration are shown in Figure 5 together with the monomer abbreviations in the caption. Indeed, these PIs are obtained using the diamines containing the electron-withdrawing –CF_3_ and –SO_2_– groups [14] and using the tetracarboxylic dianhydrides with the electron-donating –O– group. Similarly, aromatic diamines with electron-withdrawing halogenic substituents (Cl and F) directly connected to the aromatic units are also expected to be effective in reducing film coloration. However, in contrast to thermally stable CF_3_-connecting diamines such as 2,2′-bis(trifluoromethyl)benzidine (TFMB), corrosion triggered by the halogenic species, which may be yielded at elevated temperatures, is a concern.

In general, aromatic diamines with strong electron-withdrawing groups have insufficient polymerizability with tetracarboxylic dianhydrides, and it is often difficult to form ductile PI films. Film embrittlement is a fatal problem for applications as plastic substrate materials, although the toughness of transparent PI films has not been investigated significantly in the literature. A similar situation is observed in conjugated diamines including pyridine, pyrazine, and triazine units, which have a strong electron-withdrawing ability; even 2,6-diaminopyridine, which includes the pyridine unit and has the weakest electron-withdrawing power among them, often disturbs the formation of sufficiently high *M*_w_s of PAAs with film-forming ability.

As mentioned above, the incorporation of electron-withdrawing groups to tetracarboxylic dianhydrides is unfavorable because of the consequent film coloration. Nonetheless, 4,4′-(hexafluoroisopropylidene)diphthalic anhydride (6FDA), which has two electron-withdrawing CF_3_ groups, does not contribute to film coloration but significantly increases the transparency [14]. This contradiction can be attributed to the fact that the electron-withdrawing effect of the CF_3_ groups in 6FDA is inactive because the CF_3_ groups are not directly connected to the phthalic anhydride (PA) units. The increased transparency by using 6FDA probably results from the significantly inhibited intermolecular CT interactions by the bulky CF_3_ side groups and a concomitant highly distorted steric structure [25,26]. The PI derived from 6FDA and TFMB is an unusual case that results in a practically non-colored PI film [15,27]. However, unfortunately, this PI does not have a low CTE, as mentioned later [15,27].

The biphenyltetracarboxydiimide (BPDI) units in 3,3′,4,4′-biphenyltetracarboxylic dianhydride (s-BPDA)-based PIs are electronically conjugated through the biphenyl linkage. Therefore, if each phthalimide plane in the BPDI unit is almost orthogonally distorted, the coloration of the PI films can be reduced. In fact, in the isomeric 2,3,3′,4′-BPDA (a-BPDA)-based PIs, a hypsochromic and hypochromic effect in the UV-vis absorption spectrum is observed, probably owing to the biphenyl distortion arising from steric hindrance between the 2-imide C=O group and 2′-hydrogen atom at the a-BPDA-based diimide units [28].

The most effective strategy for completely removing film coloration is to use aliphatic [usually, alicyclic (cycloaliphatic)] monomers either in diamines or tetracarboxylic dianhydrides or both, whereby the CT interactions practically disappear [20,29,30,31,32,33,34,35,36,37]. However, when cycloaliphatic diamines are used, one encounters a crucial problem in the PAA polymerization process: salt formation. On the other hand, in the combination of cycloaliphatic tetracarboxylic dianhydrides and aromatic diamines, salt formation is avoidable. However, another polymerization problem emerges; cycloaliphatic tetracarboxylic dianhydrides often have insufficient polymerizability with common aromatic diamines, and, consequently, the PI films become brittle. These problems and the strategies for solving them are described later in detail.

## 3. Low-CTE Polyimides

### 3.1. Necessity of Low-CTE Materials

When a PI film closely adheres to an inorganic substrate (metals, semiconductors, and ceramics) at an elevated temperature and the obtained laminate is successively cooled to room temperature, residual stress is inevitably generated according to the following expression [38]:

(2) $$\sigma_{f}(T_{r.t.})-\sigma_{f}(T_{\mathrm{bond}})=-\int_{T_{\mathrm{bond}}}^{T_{r.t.}}\frac{E_{f}}{1-\nu_{f}}(\alpha_{f}-\alpha_{s})$$

where σ_f_ (*T*_r.t._) and σ_f_ (*T*_bond_) are the residual stresses of PI films at room temperature (*T*_r.t._) and the bonding temperature (*T*_bond_), respectively, *E*_f_ is the modulus of PI films, ν_f_ is the Poisson’s ratio of PI films, and α_f_ and α_s_ are the CTE values of PI films (commonly, 50–80 ppm·K^−1^) and inorganic substrates (e.g., ~4 ppm·K^−1^ for an amorphous silicon substrate). In general, σ_f_ (*T*_bond_) is assumed to be negligibly small. However, the stress abruptly increases in the course of cooling to room temperature because the CTE mismatch (α_f_ − α_s_) is usually large. The accumulated stress can cause significant problems, as mentioned above. However, the CTE mismatch can be reduced by choosing PI systems with low CTE values (10 ppm·K^−1^ as a provisional target).

The residual stress can also be reduced by significantly lowering the modulus of the films (*E*_f_ < 0.3 GPa). A very low modulus can be obtained by introducing a major portion of flexible siloxane blocks into the main chains [39]. However, this approach is not suitable for the present purpose because a significant decrease in the *T*_g_ is inevitable.

**Figure 6:**
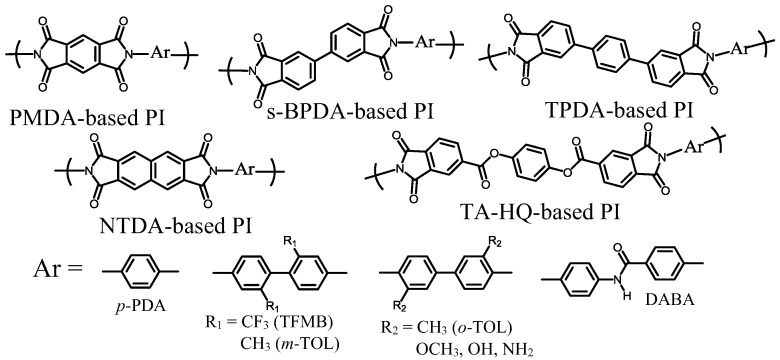
The structures of aromatic PI systems leading to low-CTE property by two-step method. PMDA = pyromellitic dianhydride, TPDA = 3,3″,4,4″-*p*-terphenyltetracarboxylic dianhydride, NTDA = 2,3,6,7-naphthalenetetracarboxylic dianhydride, TA-HQ = hydroquinone bis(trimellitate anhydride), *p*-PDA = *p*-phenylenediamine, *m*-TOL = *m*-tolidine, o-TOL = *o*-tolidine, DABA = 4,4′-diaminobenzanilide.

Figure 6 shows the structures of typical low-CTE PIs. These systems have highly linear backbone structures without exception. The low-CTE values of these PI films are associated with the highly aligned PI main chains in the *X*–*Y* direction (a high degree of in-plane chain orientation), which occurs during the thermal imidization of the PAA cast films formed (fixed) on a substrate [40,41]. Furthermore, the extent of in-plane orientation, which is closely related to the thickness-direction birefringence (∆*n*_th_ = *n*_xy_ − *n*_z_), strongly depends on the overall backbone linearity/rigidity of the PIs [42,43,44,45,46,47,48,49]. Therefore, for obtaining a low CTE, it is necessary to choose monomers with linear/rigid structures both in the tetracarboxylic dianhydrides and diamines. Drawn two-dimensionally, the repeating units of the 6FDA/TFMB system also seem to have a relatively linear structure, as shown in Figure 5. However, it is most likely that the 6FDA-based diimide (6FDI) units have no coplanarity; the phthalimide units of 6FDI are sterically twisted each other at a “hinge” portion (the hexafluoroisopropylidene linkage) [25,26]; thus, the PI main chains lose their overall linearity. Indeed, the thermally imidized 6FDA/TFMB film does not have a low CTE [15,27] (Table 1, sample 2). The situation is similar in the systems using s-ODPA and 3,3′4,4′-biphenylsulfonetetracarboxylic dianhydride (DSDA), which contain a hinge structure. A cast film from a PI solution for 6FDA/TFMB does not have a low CTE either, which is a normal phenomenon, as often observed in common polymer systems. This reflects the fact that the simple casting process using PI solutions usually does not have sufficient driving force to result in a high level of in-plane chain orientation.

On the other hand, an ether-bridged form of 6FDA, which has a coplanar structure, has been proposed with the aim of reducing both the CTE and the dielectric constant (*ε*′) [52,53]. The combination of this monomer with TFMB leads to a thermally imidized PI film that has a very low CTE (6 ppm·K^−1^), an enhanced *T*_g_, and a low *ε*′, although a reduction in the solubility is not avoidable [54]. The effect of this approach on the film coloration has not been reported in the literature.

The rod-like structure of TFMB, which includes electron-withdrawing CF_3_ groups, is expected to be useful for achieving both high transparency and low CTE. However, in fact, it is not easy to fulfill these target properties simultaneously even when TFMB is combined with rigid tetracarboxylic dianhydrides; for example, the PMDA/TFMB system (PMDA = pyromellitic dianhydride) leads to a PI film with a very low CTE (a negative value) [27,46,50]. However, the film is intensely colored [46] (Table 1, sample 1). There is a similar dilemma in the NTDA/TFMB system (NTDA = 2,3,6,7-naphthalenetetracarboxylic dianhydride) [46]. On the other hand, the s-BPDA/TFMB system affords an only slightly colored PI film [46]. In addition, it is highly possible that the s-BPDA/TFMB system results in low-CTE characteristics, as expected from its relatively linear chain structure (Figure 6). In this regard, s-BPDA/TFMB is a very limited system that has the potential to simultaneously achieve relatively high transparency and low CTE among the aromatic PI systems obtained from the commercially available monomers. However, the thermally imidized film had a CTE (34 ppm·K^−1^ [45,46]) (Table 1, sample 3) that is higher than expected, although it is most likely possible to further decrease the CTE by optimizing the film preparation conditions. The annealing of the s-BPDA/TFMB film above the *T*_g_ (314 °C [45,46]) yields an appreciably turbid film, probably owing to crystallization. Such film clouding behavior is undesirable for applications to plastic substrates.

A commercially available rod-like diamine, 2,2′,5,5′-tetrachlorobenzidine (TCB), which contains electron-withdrawing chlorine substituents, is also expected to have a similar feature to TFMB. Thermal imidization of s-BPDA/TCB leads to a highly transparent film as in s-BPDA/TFMB, and the s-BPDA/TCB film shows a moderate level of CTE (44 ppm·K^−1^) [55], which is somewhat higher than that of s-BPDA/TFMB.

### 3.2. Points to Note in the CTE Measurements

Among many evaluation points for the development of plastic substrate materials, in particular, the measurements of CTE require careful procedures. PI films formed on substrates through thermal imidization or solution casting usually accumulate residual strain at room temperature, even after they are peeled away from the substrates. This trend is more prominently observed in less thermoplastic PI systems with rigid chain structures. Before the CTE evaluation by thermomechanical analysis (TMA), the residual stress must be completely removed by annealing the films in a free state above the used imidization temperature (or final heating temperature) for less thermoplastic systems. On the other hand, for thermoplastic systems, the annealing is carried out just below the *T*_g_ to avoid significant film deformation (at a 10–20 °C lower temperature than the *T*_g_). If this pre-treatment is skipped, the specimens often undergo irreversible contraction accompanied by the release of the residual strain in the course of heating for the CTE measurements. Consequently, the TMA curve is deformed. The proper TMA curves usually consist of two straight lines with a small slope in the glassy region and an abruptly increased slope above the *T*_g_. The above-mentioned film contraction behavior often causes an “underestimation” of the magnitudes of CTE. In principle, the TMA curve in the glassy region is completely reversible between the heating and cooling processes [50]. Thus, the reversibility is direct evidence for the correctness of the CTE values. The other factors [(2)–(7)] listed below also cause similar irreversible film contraction during the CTE measurements:

(1) Release of residual strain.
(2) Desorption of adsorbed water.
(3) Evaporation of the residual solvents.
(4) Imidization of the non-cyclized portion on using imperfectly imidized samples.
(5) Crystallization.
(6) Crosslinking reactions.
(7) Thermal decomposition.

Inevitably, PI films adsorb moisture (1–3 wt % for many aromatic PIs). Therefore, to remove an undesirable influence of the adsorbed water, a set of procedures are usually conducted as follows: while maintaining a dry nitrogen flow in the TMA chamber, the preliminary first heating run of up to 120–150 °C is carried out, followed by cooling to room temperature, and re-setting to the zero elongation. Subsequently, the CTE values are determined from the TMA curves obtained in the second heating run. In addition, it is also important to choose the materials of the sensor parts in TMA (quartz or stainless steel) appropriately. Thus, the CTE values should be very carefully determined.

It is desirable to obtain not only the CTE values but also some information on the extent of the in-plane chain orientation to support the validity of the obtained results because there is a close correlation between these properties. The extent of the in-plane orientation has been estimated by various techniques such as wide-angle X-ray diffractometry [56,57], an optical waveguide method [58], polarized UV-vis absorption spectroscopy using a dichroic molecular probe [40,43,50,59], and attenuated total reflection Fourier-transform infrared spectroscopy [58,60].

The ∆*n*_th_ values can be useful to monitor the extent of the in-plane orientation using an Abbe refractometer. Previous studies have reported that there is a good correlation between the CTE and ∆*n*_th_ for wholly aromatic PIs [40,41]. Similarly, for a variety of semi-cycloaliphatic PI systems, a relatively good correlation is observed between them [20,21,36,37], even though, strictly speaking, it is difficult to compare the extent of the in-plane orientation between different systems because the intrinsic birefringence (∆*n*_0_) depends on the chain structures [61]. Therefore, the comparisons can be conducted within a fixed type of PIs (e.g., semi-cycloaliphatic PIs derived from cycloaliphatic tetracarboxylic dianhydrides and aromatic diamines) under the assumption that the ∆*n*_0_ values do not significantly differ each other. Empirically, low-CTE films (CTE < 20 ppm·K^−1^) usually have high ∆*n*_th_ values (roughly speaking, >0.05–0.1 for semi-cycloaliphatic PIs), reflecting the high degree of in-plane orientation. When a contradiction is observed between the estimated CTE and ∆*n*_th_ values, [e.g., the CTE is low (<20 ppm·K^−1^), but the ∆*n*_th_ is also low (<0.01–0.02)], there is the possibility that the CTE measurements are affected by the non-essential factors mentioned above.

## 4. Approaches to Simultaneously Achieve High Transparency, Low CTE, and Other Required Properties

### 4.1. A Transition of Development of Low-CTE, Transparent PIs

**Figure 7:**
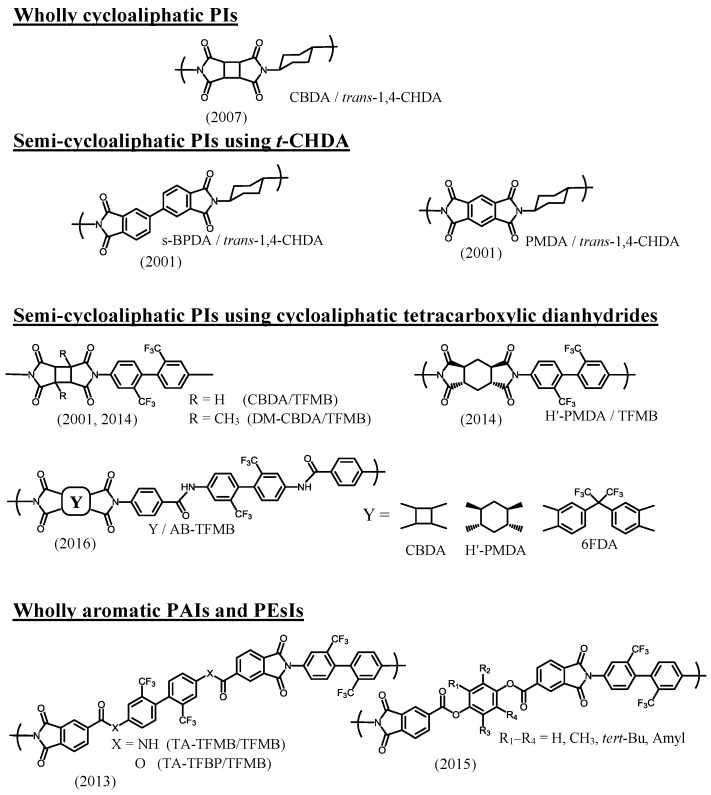
A transition for the development of low-CTE transparent PIs.

Figure 7 exemplifies the optically transparent and low-CTE PI systems that we have developed so far. In addition, we have studied solution-processable, colorless polybenzoxazole (PBO) systems [62,63], but these are not described here in detail. The optically transparent low-CTE PIs can be classified into four categories: a wholly cycloaliphatic PI (CBDA/*t*-CHDA) [17], semi-cycloaliphatic PIs derived from rigid tetracarboxylic dianhydrides and *t*-CHDA [17,45], semi-cycloaliphatic PIs derived from cycloaliphatic tetracarboxylic dianhydrides with rigid structures and aromatic diamines [36,45,51], and wholly aromatic PEsIs [21] and poly(amide imide) (PAI) [15]. The selected properties of these systems are summarized in Table 1. The detailed problems that we have encountered in these systems will be discussed later.

### 4.2. PIs Derived from Cycloaliphatic Diamines

#### 4.2.1. Problems in the PAA Polymerization

**Figure 8:**
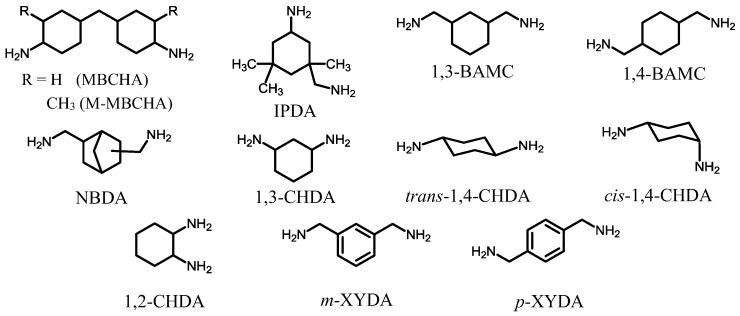
Commercially available aliphatic diamines.

**Figure 9:**
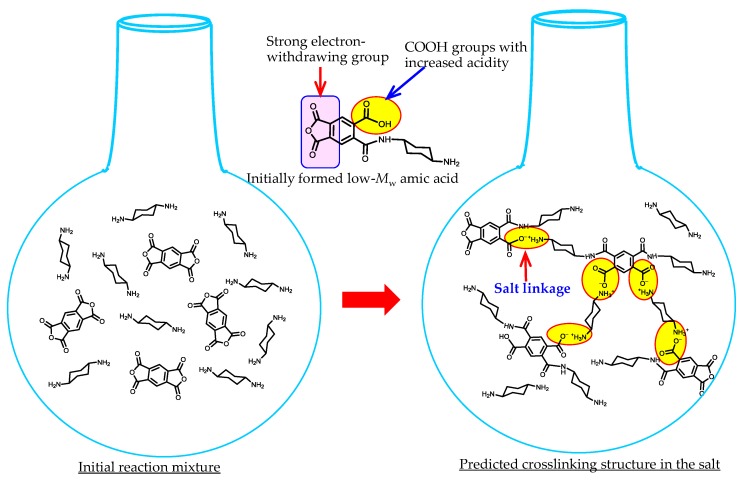
A schematic illustration of salt formation in the initial stage of PAA polymerization in anhydrous amide solvents. The PMDA/*t*-CHDA system is shown here as an example.

Commercially available aliphatic diamines with cyclic structures, which are applicable for the present purpose, are shown in Figure 8. Linear (non-cyclic) aliphatic diamines (e.g., 1,6-hexanediamine) are removed here because of the absence of sufficient heat resistance in the resultant PIs. In principle, the use of these cycloaliphatic diamines will result in colorless and heat-resistant PI films. However, the polyaddition between these diamines and tetracarboxylic dianhydrides is disturbed by the salt formation that occurs in the initial reaction stage. The formed salt probably consists of a crosslinked structure through acid–base salt bonding, as schematically depicted in Figure 9. The basicity of cycloaliphatic diamines is not significantly affected by their structures. However, in fact, the “tightness” of the formed salt strongly depends on them, as mentioned later. The salt is poorly soluble in anhydrous amide solvents, so that precipitation occurs during the polyaddition. In some cases, the PAA polymerization reactions are completely terminated, as typically observed in the PMDA/*t*-CHDA system. This system is expected to lead to a low-CTE PI film because, as well as PMDA, *t*-CHDA has high structural linearity. However, it is challenging to obtain a homogeneous PAA solution in this system owing to the formation of an extremely “tight” salt. This is probably related to (1) the shorter molecular length (a consequent higher crosslinking density) than that of other cycloaliphatic diamines, (2) the relatively planar/rigid structure of *t*-CHDA (a decrease in the solubility of the yielded salt), and (3) the enhanced COOH acidity (an intensification in the salt linkages) induced by the adjacent electron-withdrawing acid anhydride group in the low-*M*_w_ amic acids, as shown in Figure 9. The difficulty in the polyaddition between PMDA and *t*-CHDA does not change, even after dilution and heating [17]. A similar situation is also observed in the reaction between CBDA and *t*-CHDA [17].

On the other hand, when flexible cycloaliphatic diamines (e.g., 4,4′-methylenebis(cyclohexylamine) (MBCHA, a mixture of isomers, Figure 8)) were used, the situation is different from the case using *t*-CHDA. For example, in the reaction of PMDA and MBCHA, a salt is similarly formed in the initial reaction stage, but it is gradually dissolved by simply stirring at room temperature for a prolonged period, and, finally, a homogeneous/viscous PAA solution is formed. This is probably due to the decreased crosslinking density in the salt resulting from the use of MBCHA with a distorted and long molecular structure compared to *t*-CHDA [method (a) listed below]. Consequently, the predicted “loosely formed” salt permits the penetration of the solvent molecules into the salt, which helps the dissolution of the salt. Similar behavior is also observed in the systems using isophoronediamine (IPDA), 2,5(2,6)-bis(aminomethyl)bicyclo[2.2.1]heptane (NBDA), and other flexible cycloaliphatic diamines shown in Figure 8. However, even when these flexible cycloaliphatic diamines are chosen, a crucial problem remains, i.e., the insufficient reproducibility with respect to the *M*_w_s of the resultant PAAs (or more conveniently, their inherent viscosities, η_inh_). This is a great obstacle particularly concerning mass-production for the commercialization of semi-cycloaliphatic colorless PIs using cycloaliphatic diamines.

To monitor the *M*_w_-reproducibility, the measurements of the reduced viscosity (η_red_) of PAAs at a fixed low polymer concentration (usually, 0.5 wt %), for example, by an Ostwald viscometer is often more useful in terms of speed than gel permeation chromatography (GPC). That is because PAAs decompose with a prompt decrease in the *M*_w_ as soon as the PAA solutions are diluted to a low concentration (e.g., 0.05 wt % as for GPC measurements). Thus, the η_red_ measurements can be carried out much more rapidly than GPC. In addition, it is not necessary to determine the inherent viscosity (η_inh_) by extrapolation to zero concentration because the extrapolation is disturbed by an unusual increase in the η_red_ values in the low concentration range by the polyelectrolyte effect. Therefore, the η_red_ values measured at 0.5 wt % can be regarded, for all practical purpose, as the η_inh_ values. This parameter is also useful for soluble PI systems; however, it is difficult to obtain the viscosity data after imidization for PIs using cycloaliphatic diamines owing to their insolubility. The η_inh_ values give a useful criterion; PIs with a η_inh_ value lower than 0.3–0.5 dL·g^−1^ often have an insufficient film-forming ability. When the PIs of interest are highly soluble, it is desirable to conduct not only the GPC measurements for the PIs but also a set of viscosity measurements for both the PAAs and the corresponding PIs because an undesirable molecular weight decrease during imidization, if any, can be monitored by comparing between these η_inh_ values.

Other methods [(b)–(e)] for preventing or suppressing the salt formation have also been reported:

(a) Choice of higher-*M*_w_ cycloaliphatic diamines with bulkier and more distorted structures.
(b) Choice of a higher-*M*_w_ tetracarboxylic dianhydride with bulkier and more distorted structures.
(c) Optimization of the reaction conditions.
(d) One-pot polymerization method (only for highly soluble PI systems).
(e) Addition of acetic acid.
(f) Use of silylated cycloaliphatic diamines.

**Figure 10:**
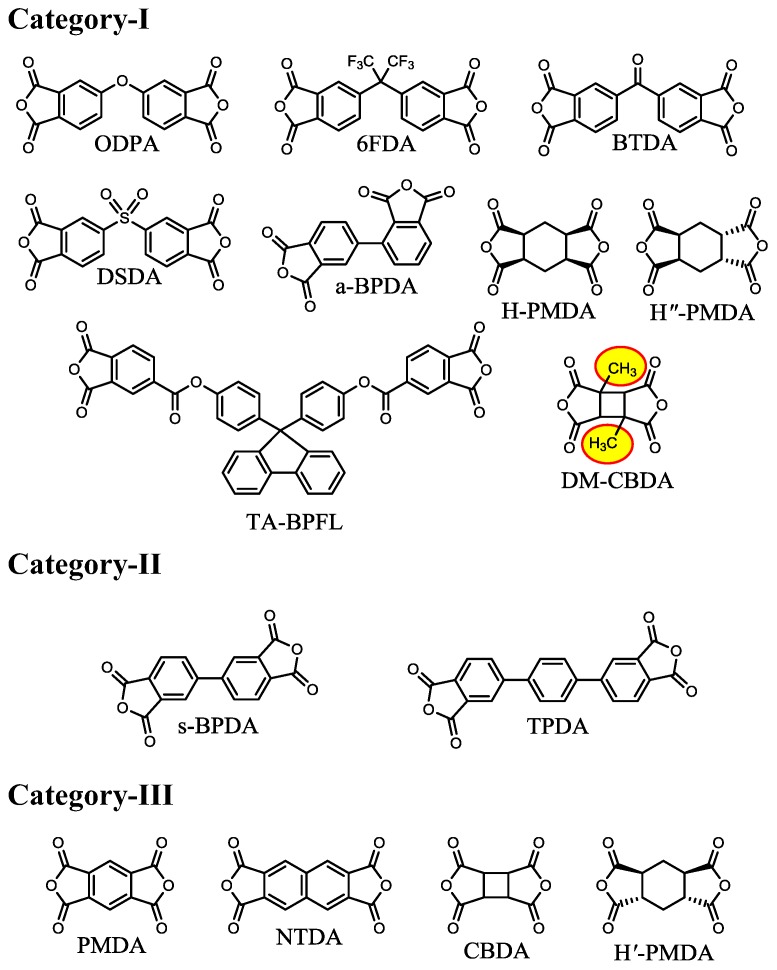
Classification of tetracarboxylic dianhydrides on the basis of the polymerizability with *trans*-1,4-CHDA in DMAc. Reproduced with permission from Elsevier [37].

Here, method (b) has a similar effect to method (a). For example, in a rare case, a sulfone-containing tetracarboxylic dianhydride (DSDA, Figure 10) reacts smoothly with *t*-CHDA, as in the use of aromatic diamines, without prominent salt precipitation. A cardo-type tetracarboxylic dianhydride (TA-BPFL, Figure 10) possessing a bulky fluorenyl side group is also useful in preventing the formation of a tight salt [64]. Incidentally, the molecular bulkiness of TA-BPFL also contributes to the excellent solubility of the resultant PIs; surprisingly, the thermally imidized TA-BPFL/*t*-CHDA film maintains excellent solubility in contrast to the fact that *t*-CHDA-derived PIs are usually quite insoluble in common organic solvents [64]. Method (c) also sometimes contributes to the rapid dissolution of the salt by optimizing the reaction conditions [the monomer content, the type of solvents, and reaction temperature]. An unusual monomer feeding procedure (the addition of diamine to tetracarboxylic dianhydride solutions [65]) is also a possible method, although it is not always effective in accelerating the salt dissolution. However, this method is not suitable in terms of enhancing the *M*_w_s of the PAAs because the tetracarboxylic dianhydrides dissolved in advance suffer partial hydrolysis by a small quantity of water contained in the solvents. Method (d) is applicable when the imidized forms are highly soluble, and it is expected to be effective in suppressing salt formation.

Method (e) has some effect on shortening the reaction period [66]. In this case, the added acetic acid probably acts as an end-capping reagent for the unreacted aliphatic amino groups that are the origin of crosslinking via salt linkages (Figure 9).

In principle, the silylation method (f) can completely inhibit salt formation because the reactions between trialkyl-silylated cycloaliphatic diamines and tetracarboxylic dianhydrides yield the trialkylsilylester forms of PAAs, which do not contain the COOH groups necessary for salt formation [67]. However, in our experience, even these methods ((e) and (f)) are not always universally effective because there are several systems where no homogeneous precursor solutions are obtained.

At present, *t*-CHDA is a very limited cycloaliphatic diamine, suitable for achieving simultaneously excellent transparency, high *T*_g_, and low-CTE characteristics. In contrast, isomeric *cis*-CHDA, as well as as-hydrogenated *p*-PDA (a mixture of the *trans*- and *cis*-isomers), is not useful for obtaining a low CTE because of the significant decrease in the chain linearity on using the *cis*-form.

We have also found that the reaction behavior between *t*-CHDA and a variety of tetracarboxylic dianhydrides in DMAc can be classified into three categories based on the “tightness” of the initially formed salt, as shown in Figure 10. The results correspond well to the assumption mentioned above that the “tightness” of the salt is dominated by the structural rigidity and bulkiness of the monomers used, the strength of the salt linkages, and the predicted crosslinking density in the salt. The tetracarboxylic dianhydrides in category-I result in homogeneous PAA solutions after prolonged stirring at room temperature via gradual dissolution of the initially formed salt. These tetracarboxylic dianhydrides commonly have distorted or bulky structures, corresponding to method (b) mentioned above. However, the systems using semi-rigid tetracarboxylic dianhydrides belonging in category-II require heating to obtain homogeneous PAA solutions because of the formation of a relatively tight salt. For example, in the s-BPDA/*t*-CHDA system, the salt-containing reaction mixture in DMAc was heated at 100–150 °C for a very short period (< a few minutes) to induce partial dissolution of the salt in association with an exothermic reaction. The reaction mixture was stirred without additional heating, using only the heat of reaction, until the reaction mixtures homogenized. This set of processes yield a high-*M*_w_ PAA (η_inh_ = 1.5–2.5 dL·g^−1^) [45]. A solvent effect was also observed in this system; for example, the replacement of DMAc by NMP enables the formation of a homogeneous PAA solution by continuous stirring at room temperature (without heating), probably owing to slightly increased salt solubility in NMP. However, the use of NMP is not desirable because it tends to cause slight coloration in the resultant PI film compared to DMAc, as mentioned above. On the other hand, the systems in category-III are not polymerizable under any conditions [17,37,46]. The difficulty in the PAA polymerization probably reflects the formation of an extremely tight salt, which corresponds well to the extremely rigid monomer structures in category-III. A comparison between the CBDA/*t*-CHDA system (category-III) and the 1,3-dimethyl-CBDA (DM-CBDA)/*t*-CHDA system (category-I) suggests that the dimethyl substituents (highlighted in Figure 10) play a great role in reducing the tightness of the salt. Thus, the promising systems (category-III) in terms of the target properties inevitably involve polymerization problems.

#### 4.2.2. Properties of *t*-CHDA-Derived PIs

Even though the category-III systems require industrially incompatible processes (toxic and high-cost), it is important to ascertain the potential of these systems from the viewpoint of material science. The CBDA/*t*-CHDA polyimide can be produced with great difficulty; the PI precursor can be polymerized only under very limited conditions (the combination of the partial silylation of *t*-CHDA and the use of a mixed solvent of DMAc and toxic HMPA) [17]. The precursor cast film on the substrate was immersed in a methanol bath to prevent the significant film coloration induced by residual HMPA during thermal imidization. This extraction process permits the desilylation and the complete removal of the residual solvent while maintaining good adhesion between the film and the substrate. The subsequent thermal imidization leads to a good-quality transparent PI film with a relatively low CTE [17], as listed in Table 1 (sample 4). Similarly, the PMDA/*t*-CHDA system requires a similar complicated process to obtain a good-quality PI film. The resulting PI film displays a further decreased CTE (Table 1, sample 6), reflecting its higher chain linearity [17].

On the other hand, as mentioned above, the systems in category-II are compatible with the conventional two-step process. Furthermore, the resultant PI films show excellent combined properties. For example, the s-BPDA/*t*-CHDA film has high optical transparency (*T*_400_ = ~80%), a high *T*_g_ (>300 °C), and a very low CTE (10 ppm·K^−1^ [45]) (Table 1, sample 5). Thus, at present, s-BPDA/*t*-CHDA seems to be the most practically valuable system of the *t*-CHDA-derived PIs in terms of its simple manufacturing processes and the excellent properties mentioned above. However, there is still room for improvement in the film ductility because this PI film was somewhat brittle (the maximum elongation at break in the tensile test, *ε*_b max_, was about 10%). This is probably inevitable because of the predicted poor chain entanglement arising from its linear/stiff chain structure. Our attempts extend to improving the film ductility while maintaining the very low CTE. Copolymerization with flexible monomers is often effective in improving the film ductility of s-BPDA/*t*-CHDA. However, the CTE of the copolymers inevitably increases with increasing flexible comonomer contents (our unpublished results). Thus, it is very difficult to escape from this “additivity rule” between the CTE and the copolymer composition. Similarly, s-BPDA/*t*-CHDA can be modified by sequence-controlled copolymerization with a flexible cycloaliphatic diamine (NBDA, Figure 8), accompanied by an increase in the CTE with increasing NBDA content [68].

### 4.3. PIs Derived from Cycloaliphatic Tetracarboxylic Dianhydrides

#### 4.3.1. Problems in the Polymerizability of Cycloaliphatic Tetracarboxylic Dianhydrides with Aromatic Diamines and other Problems

**Figure 11:**
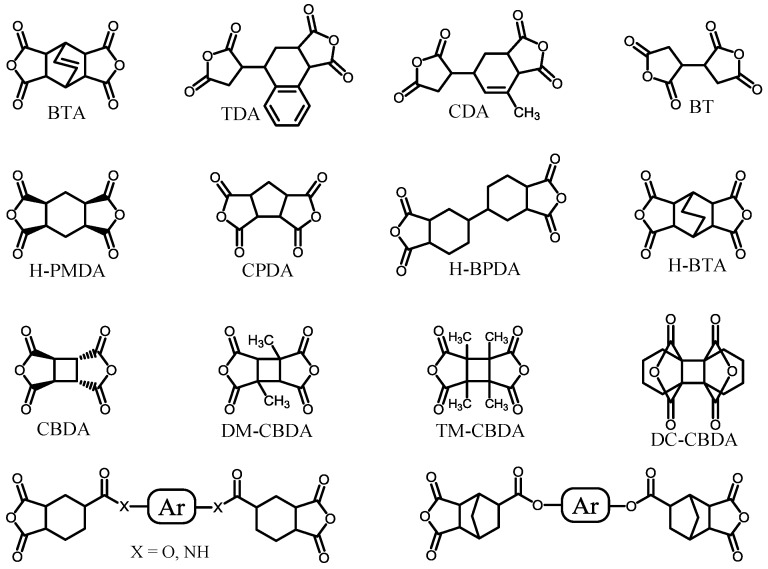
Commercially available cycloaliphatic tetracarboxylic dianhydrides (two monomers shown in the bottom are those prepared by the author’s group).

**Figure 12:**
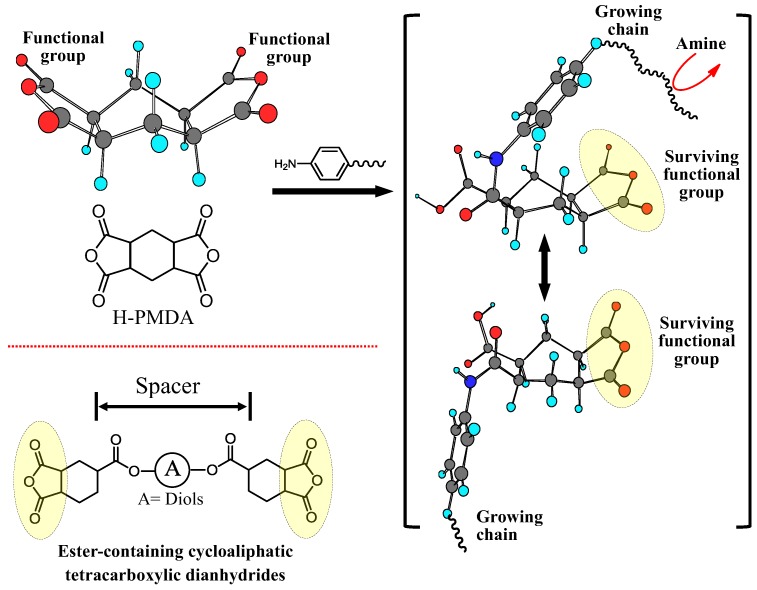
A self-steric hindrance model for explaining the η_inh_-based H-PMDA polymerizability much lower than that of PMDA and ester-inked HTA-derived cycloaliphatic tetracarboxylic dianhydrides. Reproduced with permission from Elsevier [35].

The polymerizability for obtaining higher *M*_w_s of PAAs is a very important factor because it is directly connected to the PI film ductility. The reactions between cycloaliphatic tetracarboxylic dianhydrides and aromatic diamines proceed smoothly without salt formation. Consequently, PAAs are obtained with good reproducibility concerning their *M*_w_s. A variety of cycloaliphatic tetracarboxylic dianhydrides have been synthesized on the laboratory scale [13,16,29,31,33,35,36,69,70,71,72,73]. Figure 11 shows cycloaliphatic tetracarboxylic dianhydrides that are commercially available, except for two monomers shown in the bottom of Figure 11. However, many of these cycloaliphatic tetracarboxylic dianhydrides do not have sufficient polymerizability with aromatic diamines, and they have also problems for low-cost mass productivity. For example, BTA (Figure 11) does not lead to ductile films because of the insufficient *M*_w_s (or η_inh_) of the obtained PAAs, even when combined with highly reactive common aromatic diamines, as suggested from the very low η_inh_ value of 0.19 dL·g^−1^ for BTA/4,4′-ODA) [35]. Hydrogenated PMDA (H-PMDA, Figure 11) is inferior to pristine PMDA in terms of the polymerizability [17,35], which can be conveniently estimated by the η_inh_ values of PAAs, as suggested by the fact that η_inh_ = 0.60 dL·g^−1^ for H-PMDA/4,4′-ODA and η_inh_ > 2 dL·g^−1^ for PMDA/4,4′-ODA. To explain the decreased η_inh_-based polymerizability of BTA and H-PMDA compared with common aromatic tetracarboxylic dianhydrides, we have proposed a model based on their steric structures [17,20,35,37]; H-PMDA has a steric structure consisting of the 1-*exo*, 2-*exo*, 4-*exo*, 5-*exo* configuration for the four C=O groups with the boat-form of the central cyclohexane unit [74]. BTA also has a similar all-*exo* steric structure (Information from the academic research section of Tokyo Chemical Industry Co., Ltd.).As shown in Figure 12, after the attack by an amine-terminated oligomer on one functional group of H-PMDA, as well as BTA, the adjacent growing chain can cover/shield the surviving functional group by a conformational change at the formed amide linkage, whereby other amine-terminated reactants cannot approach the surviving functional group because of a blocking effect by its own chain. When using PMDA, such “self-steric hindrance” is avoidable because the two functional groups face in the opposite direction to each other, and the growing chain is sufficiently far apart from the surviving functional group.

If the model proposed here is reasonable, inserting a spacer between the functional groups of H-PMDA, the polymerizability should be dramatically enhanced. Based on this concept, we synthesized ester-containing cycloaliphatic tetracarboxylic dianhydrides from a derivative of hydrogenated trimellitic anhydride (1-*exo*,2-*exo*,4-*exo*-cyclohexanetricarboxylic acid anhydride: HTA) and various diols such as hydroquinone (HQ) and 4,4′-biphenol (44BP) (bottom left in Figure 11). Indeed, an ester-linked cycloaliphatic tetracarboxylic dianhydride derived from HTA and HQ (i.e., HTA-HQ) shows dramatically enhanced polymerizability with various aromatic diamines, as illustrated from the high η_inh_ values of the PAAs. For example, the reaction between HTA-HQ and 4,4′-ODA results in a very high η_inh_ value of 2.3 dL·g^−1^ [35], which is much higher than the η_inh_ value (0.6 dL·g^−1^) for H-PMDA/4,4′-ODA. In addition, we confirmed that hydrogenated s-BPDA also has high polymerizability with various aromatic diamines. These results indicate that the proposed “self-steric hindrance” model is reasonable.

Among the cycloaliphatic tetracarboxylic dianhydrides shown in Figure 11, CBDA shows the highest η_inh_-based polymerizability with various aromatic diamines, comparable to PMDA [17,36,45]; for example, in the CBDA/TFMB, a PAA with a very high η_inh_ value (>2 dL·g^−1^) is easily obtained, unlike the H-PMDA/TFMB system, which has a low η_inh_ value (<0.4 dL·g^−1^) [17,35,36]. DM-CBDA is also highly reactive, although it is somewhat inferior to the substituent-free CBDA. The considerably high reactivity of CBDA, as well as DM-CBDA, can be explained by a combined effect of the “direction” of the functional groups, similar to that of PMDA, and the “strain” accumulated in the acid anhydride ring connected to the highly strained four-membered central cyclobutane ring [36]. However, surprisingly, 1,2,3,4-tetramethyl-substituted CBDA (TM-CBDA, Figure 11) is poorly reactive with aromatic diamines, and the resultant PAAs have no film-forming ability [36]. This probably results from a significant steric hindrance effect of these substituents against the attack of the amine reactants.

Thus, CBDA seems to be a perfect cycloaliphatic dianhydride for the present purpose. However, this is not always true. Because of the presence of the strain accumulated at the anhydride rings, CBDA easily undergoes the ring-opening reaction by hydrolysis. Indeed, it is very unstable in humid air. In addition, the ease of ring opening (in other words, the ease of PAA formation) by strain release suggests difficulty in the ring closure (i.e., imidization). Indeed, in our experience, to complete imidization, the CBDA-based systems require heating at higher temperatures than in other systems (e.g., H-PMDA-based systems). This is an undesirable situation for suppression of film coloration. Therefore, if possible, it is preferable to prepare the CBDA-based PI films through the casting of the PI solutions without the high-temperature thermal imidization process. However, unfortunately, this requirement is often inhibited by the fact that CBDA-based PIs are mostly insoluble in common organic solvents. Thus, for CBDA-based PI systems, there is no other choice than the conventional two-step process for PI film preparation. Our approaches to improve the solubility of CBDA-based PIs are presented later.

CBDA is synthesized via the photo-dimerization of maleic anhydride in solution [29]. Therefore, this situation is not always favorable in terms of the mass-production of CBDA, compared to common thermal reaction systems using large-scale reactors.

**Figure 13:**
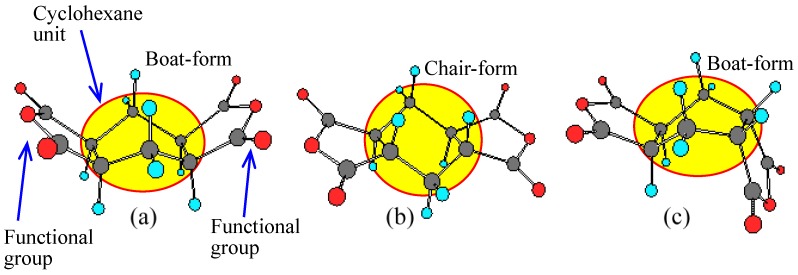
The steric structures of three isomers of hydrogenated PMDA as determined by single-crystal X-ray diffraction method: (**a**) H-PMDA, (**b**) H′-PMDA, (**c**) H″-PMDA. Reproduced with permission from Elsevier [37].

We have investigated the steric effects on the polymerizability using three H-PMDA isomers. Figure 13 shows the steric structures of these isomers, determined by the single-crystal X-ray diffraction. H″-PMDA (Figure 13c) shows much higher η_inh_-based polymerizability than H-PMDA [20]. This probably, in accordance with the above-mentioned self-steric hindrance mechanism, reflects the fact that the functional groups almost face in opposite directions to each other in H″-PMDA, reducing the self-steric hindrance.

An additional isomer, H′-PMDA (Figure 13b) also has much higher polymerizability than H-PMDA. H′-PMDA consists of a unique steric structure, where the unusually non-planar acid anhydride rings are connected to the central cyclohexane unit with the chair-form [37]. It is highly possible that the non-planar acid anhydride rings are strongly strained, as suggested by the unusual bond angles in the functional groups. In this regard, the observed high polymerizability of H′-PMDA may be related to such strain effects. The presence of the ring strain in H′-PMDA should be reflected in its thermodynamic stability. Subsequently, we estimated the standard enthalpy of formation of these isomers at 25 °C (Δ*H*_f_°_298_) from the heats of combustion (*Q*_c_), which were experimentally obtained from an advanced adiabatic bomb calorimeter. The results are Δ*H*_f_°_298_ = −8.88 × 10^2^ kJ·mol^−1^ for H′-PMDA, −1.00 × 10^3^ kJ·mol^−1^ for H-PMDA, and −1.05 × 10^3^ kJ·mol^−1^ for H″-PMDA. Indeed, H′-PMDA has appreciably lower thermodynamic stability than the other isomers (H-PMDA and H″-PMDA). The results do not conflict with the interpretation of the high polymerizability of H′-PMDA based on the ring strain [37].

#### 4.3.2. Properties of PIs Derived from Cycloaliphatic Tetracarboxylic Dianhydrides

Ester-linked cycloaliphatic tetracarboxylic dianhydrides (bottom left in Figure 11), which are synthesized from HTA derivative and diols, can be easily modified by selecting a variety of diols as the raw materials. The resultant PIs have high flexibility of molecular design because diamines for combination with HTA-derived tetracarboxylic dianhydrides are also extensively commercially available. In addition, copolymerization with other tetracarboxylic dianhydrides permits further structural modifications to optimize the target properties. For example, the combination of a cycloaliphatic tetracarboxylic dianhydride synthesized from *cis*-type HTA (all-*exo* configuration for three C=O groups with a boat-form central cyclohexane unit) and a rigid structure of 44BP with a rod-like diamine, *o*-tolidine (*o*-TOL) results in a PI film with high optical transparency (*T*_400_ = 81.6%), a high *T*_g_ (295 °C), and very high toughness (*ε*_b max_ > 100%) via chemical imidization [35]. Furthermore, this PEsI film is not only highly soluble even in less hygroscopic solvents such as cyclopentanone (CPN) at room temperature but also highly thermoplastic. However, unfortunately, even when linear/rigid structures were chosen both in the diols (e.g., HQ and 44BP) as the raw materials and the diamines (e.g., *p*-PDA and *o*-TOL), the resulting PIs did not have low CTE values [35] (Table 1, sample 9). This is probably attributed to its decreased overall main chain linearity by a sterically distorted and non-planar local structure of the *cis*-type HTA-based hydrogenated trimellitimide unit. When using another HTA isomer with a controlled steric structure as another raw material, a certain reduction in the CTE is observed in the resulting PEsIs [75].

We have also examined an analogous ester-linked cycloaliphatic tetracarboxylic dianhydride using norbornanetricarboxylic anhydride (NCA) with a bicyclic structure (bottom right in Figure 11) [76]. The resultant PIs show appreciably higher *T*_g_s than the corresponding mono-cyclic counterparts (HTA-derived systems), probably owing to the suppression of the internal rotation by the bicyclic (crosslinked) structure at the norbornane units. However, these systems also do not have low CTE values, corresponding to the fact that these PI systems commonly have similar local steric structures (*cis*-form) at the HTA- and NCA-based tricarboxyimide units. Thus, these results emphasize the importance of precisely controlling the steric structures of all the cycloaliphatic moieties included in the PI main chains.

**Figure 14:**
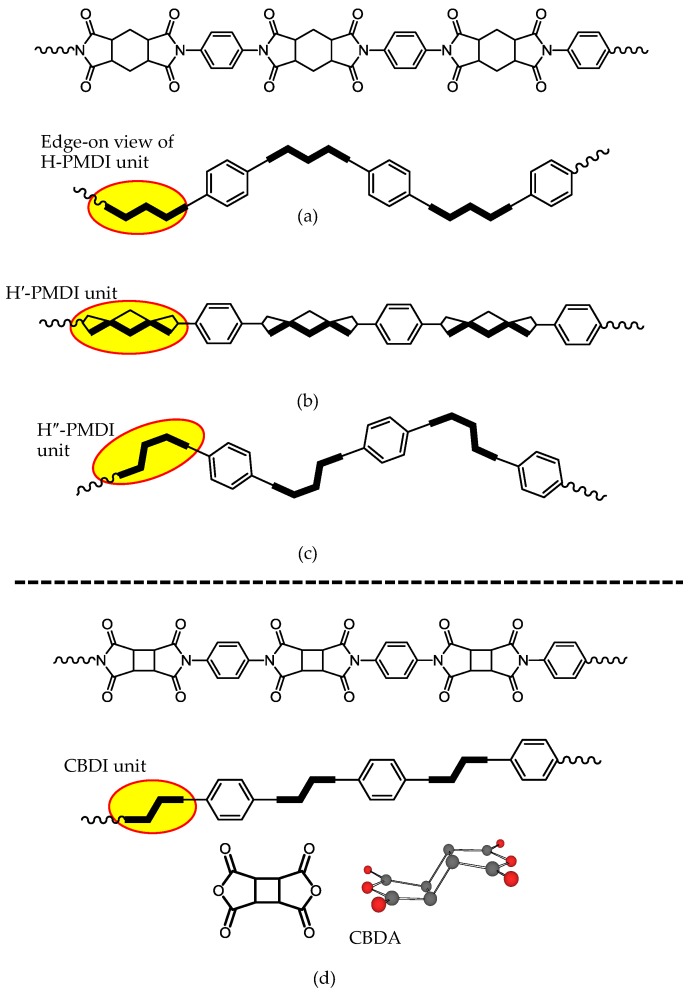
Schematic illustrations of extended PI chains and an edge-on view of the diimide units for the systems derived from H-PMDA isomers: (**a**) H-PMDA, (**b**) H′-PMDA, and (**c**) H″-PMDA, and (**d**) CBDA as a comparative system. The systems using *p*-phenylenediamine as a typical rigid diamine are shown here for simplicity. Reproduced with permission from Elsevier [37].

A similar situation is observed in the H-PMDA isomeric systems. The extended chain forms are schematically drawn in Figure 14, where an edge-on view of the diimide units (H-PMDI, H′-PMDI, and H″-PMDI) is depicted for clarity [37]. In the H-PMDA- and H″-PMDA-based PI systems, the extended chains “meander” significantly, which is inevitable because of the presence of the distorted (non-planar) local structures in their diimide moieties, even when rod-like diamines such as *p*-PDA are used. This corresponds to the fact that the H-PMDA- and H″-PMDA-based PI systems usually do not have low CTE values. Such significantly meandering chain form, particularly, in H″-PMDA-based PIs also corresponds to the outstandingly enhanced solubility of H″-PMDA-based PIs [20].

In contrast, the H′-PMDA-based PI system can take a highly linear extended chain form as shown in Figure 14b. Therefore, the use of H′-PMDA is expected to be suitable for reducing the CTE. In fact, only when combined with rigid diamines such as DABA, the resultant H′-PMDA-based PI films show distinctly decreased CTE values compared with the H-PMDA- and H″-PMDA-based counterparts [37].

The *M*_w_s of PIs and the drying conditions during the solution casting also affect the resultant CTE values somewhat; the use of higher *M*_w_s of PIs and drying under milder thermal conditions (i.e., not drying quickly at high temperatures but drying slowly using multi-step heating) contribute to an appreciable decrease in the CTE. For example, the H′-PMDA/TFMB film shows a relatively low CTE (30 ppm·K^−1^) on optimizing these conditions, in addition to the other achieved target properties such as excellent optical transparency, a very high *T*_g_ (357 °C), high toughness, and excellent solution-processability [37] (Table 1, sample 10).

On the other hand, CBDA is also expected to be useful for reducing the CTE owing to a crank-shaft-like steric structure of the CBDA-based diimide (CBDI) units (see its edge-on view in Figure 14d), which maintains the overall main chain linearity in combination with the rigid/linear structures of diamines. Indeed, the CBDA/TFMB system (Figure 7) provides a colorless PI film with a relatively low CTE (21 ppm·K^−1^) [36,45] (Table 1, sample 7), which is lower than that of H′-PMDA/TFMB. However, CBDA also tends to result in some undesirable properties; CBDA-based PIs are mostly insoluble in common organic solvents (i.e., the PIs do not possess solution-processability and the chemical imidization process compatibility), and they are often brittle (e.g., *ε*_b_ < 10% for CBDA/TFMB) [51]. The DM-CBDA/TFMB system (Table 1, sample 8) has similar properties to CBDA/TFMB.

**Figure 15:**
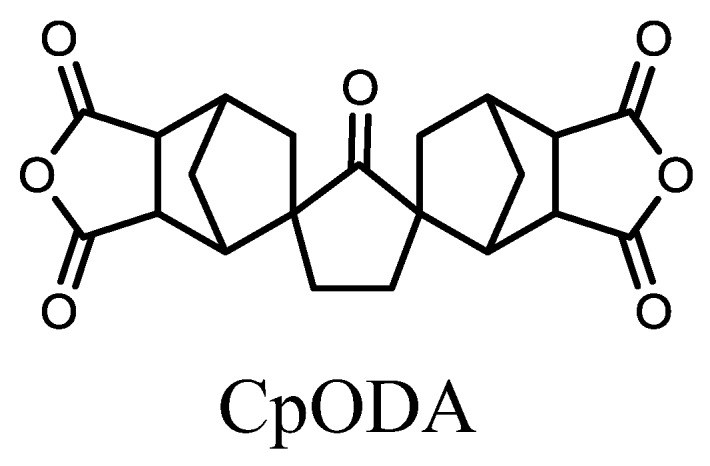
The structure of cycloaliphatic tetracarboxylic dianhydride with cyclopentanone bis-spironorbornane structure (CpODA) [77].

A novel cycloaliphatic tetracarboxylic dianhydride with a cyclopentanone bis-spironorbornane structure (CpODA, Figure 15) has been reported [77]. Even when combined with the rigid diamine *m*-tolidine (*m*-TOL), the PI obtained via chemical imidization is highly soluble, which can be attributed to the bulky and twisted steric structure of CpODA, where the NCA plane is orthogonally connected to the CPN plane. For the CpODA/*m*-TOL system, solution casting after chemical imidization results in a colorless PI film with a somewhat low CTE (38 ppm·K^−1^) and a high *T*_g_ (323 °C) [77]. The observed high *T*_g_ probably results from inhibited internal rotation between the central CPN and the NCA units, which arises from the spiro structure of CpODA.

Concerning our target properties, while the optical transparency, *T*_g_, CTE, and solubility are properties determining practical use, the film toughness is also important. Despite this, in the literature, the film toughness has not always been examined. This might be because cycloaliphatic PIs often yield brittle films, and it is not easy to overcome the embrittlement problem by processing alone. The strategies for solving this issue are also currently under consideration [78,79].

### 4.4. Cycloaliphatic PIs Showing High Transparency, Low CTE, and Sufficient Ductility by Simple Drying of Coated PI Solutions

#### 4.4.1. A Favorable Property Accompanying the Solubility Improvement of PIs

**Figure 16:**
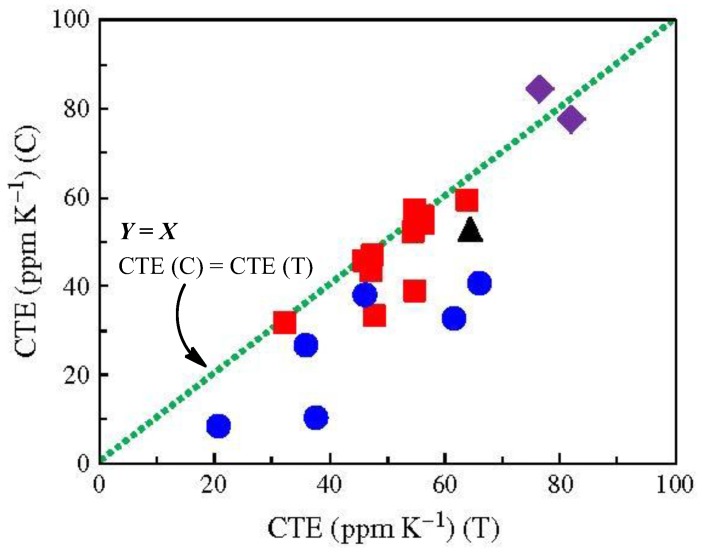
The effect of imidization method [chemical (C) and thermal (T) imidization processes] on CTE. (▲) 6FDA/TFMB, (♦) HTA-HQ-based systems, (■) H″-PMDA-based systems, and (●) TA-TFMB-based systems. Reproduced with permission from John Wiley & Sons [51].

As mentioned above, compatibility with the chemical imidization process is the key processing factor for suppressing PI film coloration. We also found out that this influences the resultant CTE values [15]. Figure 16 shows an effect of the imidization methods [chemical imidization (C, ordinate) and thermal imidization (T, abscissa)] on the CTE of the resultant PIs that we have studied so far. The results show that the plots are mostly positioned in the region below the *Y* = *X* line (the deviation from this line is the largest for TA-TFMB/TFMB), indicating that the film preparation method via chemical imidization is superior to the conventional two-step process via thermal imidization in terms of reducing the CTE values [15,21]. Recall that chemical imidization process is also advantageous for enhancing the optical transparency of the PI films (Figure 3). Thus, these results suggest that compatibility with the chemical imidization process holds the key to success.

The fact that the PI films prepared via chemical imidization have lower CTE values than those via the two-step process means that there exist some specific PI systems where simple solution casting induces more prominent “spontaneous” in-plane chain orientation [15,20,21,36,37,51]. However, the detailed mechanism of this phenomenon is not clear at present. That is because it seems that the simple solution casting process involves no effective driving forces for causing a high degree of in-plane orientation, as suggested by the fact that cast films of common polymers usually have an almost three-dimensionally random orientation distribution (e.g., in our experience, NMP-cast PES film has practically zero ∆*n*_th_ value).

#### 4.4.2. An Approach to Improve the Poor Solubility of CBDA-based PIs while Maintaining Their High Transparency and Low-CTE Property

**Figure 17:**
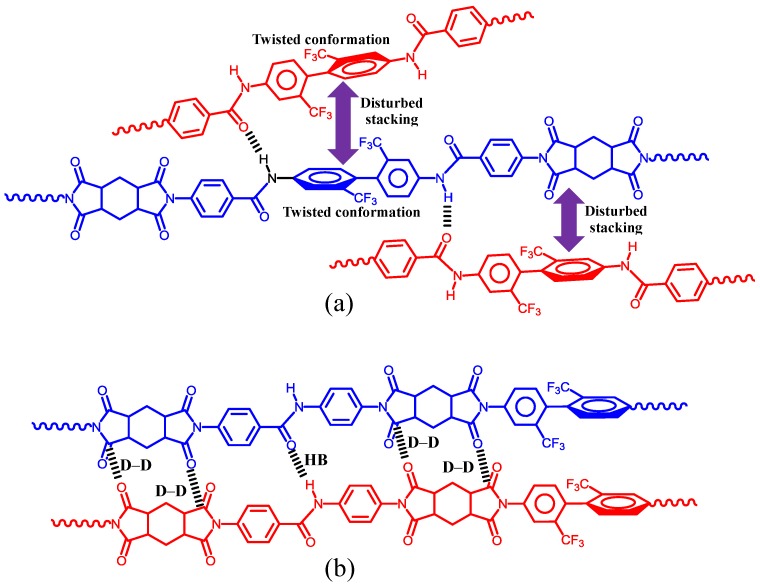
A schematic sketch expressing the difference of interchain interactions [hydrogen bonding (HB) and dipole–dipole (D–D) interaction between the imide C=O groups]: (**a**) H′-PMDA/AB-TFMB and (**b**) H′-PMDA/TFMB;DABA copolymers. Reproduced with permission from John Wiley & Sons [51].

DABA is often superior to TFMB in terms of reducing the CTE values of PI films. Our initial attempt was to further decrease the CTE of H′-PMDA/TFMB by copolymerization with DABA while maintaining the compatibility with the chemical imidization process [51]. However, the use of DABA as a comonomer causes gelation during the chemical imidization process even at a low DABA content of 30 mol %. This is attributed to the significantly decreased solubility of the copolyimides using DABA. Thus, our initial attempt, the modification of H′-PMDA/TFMB with DABA, was unsuccessful. Subsequently, we synthesized a novel diamine (AB-TFMB, Figure 7), which has the structural features of TFMB and DABA. Favorably, this diamine is obtained without coloration in contrast to highly colored DABA. The combination of H′-PMDA and AB-TFMB leads to a PI system compatible with the chemical imidization process because of its dramatically improved solubility. Despite the significantly increased amide content in H′-PMDA/AB-TFMB compared to that in the above-mentioned copolyimide [i.e., H′-PMDA/TFMB(70);DABA(30)], the dramatically enhanced solubility in H′-PMDA/AB-TFMB can be explained by the effectively disturbed interchain amide–amide hydrogen bonds in the 2,2′-CF_3_-assisted highly twisted biphenylene units, which are positioned adjacent to the amide linkages [51], as schematically depicted in Figure 17a. In contrast, as shown in Figure 17b, the amide–amide hydrogen bonds in the H′-PMDA/TFMB;DABA copolymers remain active in addition to the presence of the possible dipole–dipole attractive interactions between the imide C=O groups [80,81], which have been accepted as the origin of strong intermolecular forces in PIs. Thus, the great difference in the solubility between H′-PMDA/AB-TFMB and H′-PMDA/TFMB;DABA can be reasonably explained by this hypothesis. Another merit of using AB-TFMB is that the H′-PMDA/AB-TFMB film prepared via chemical imidization is non-colored, ductile, and has a relatively low CTE of 25 ppm·K^−1^ [51] (Table 1, sample 11).

On the other hand, the replacement of H′-PMDA by CBDA for combination with AB-TFMB is expected to be effective in further decreasing the CTE. However, this attempt caused a significant decrease in the solubility and, as a result, disturbed the chemical imidization process. A modification of CBDA/AB-TFMB by copolymerization using 6FDA (30 mol %) results in a sufficiently ductile (*ε*_b max_ > 30%) and colorless PI film (*T*_400_ > 80%) with an ultra-low CTE of 7.3 ppm·K^−1^ [51] (Table 1, sample 12). This is very surprising because copolymerization using 6FDA usually causes a significant increase in the CTE, owing to the highly distorted structure of 6FDA. Even at a lower 6FDA content (20 mol %), the film preparation process via chemical imidization could be used, and the cast film displayed a further decreased CTE value (4.2 ppm·K^−1^) [51] (Table 1, sample 13), which is close to that of silicon wafers. However, the haze appreciably increased (from 1.5% at 30 mol % of 6FDA to 3.4% at 20 mol % of 6FDA), probably owing to a sort of micro-heterogeneous chain flocculation phenomenon during solution casting, arising from the reduction in solubility.

**Figure 18:**
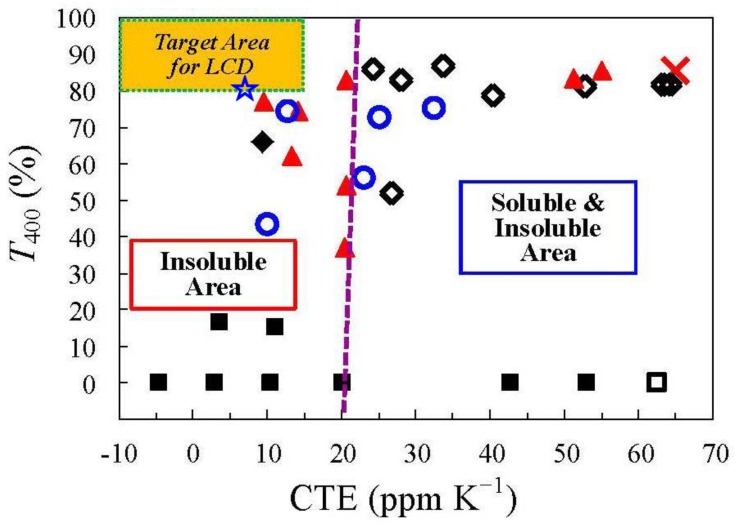
CTE–transparency (*T*_400_)–solubility diagram for selected PI systems that we have investigated so far. Open and closed symbols denote soluble (compatible with the chemical imidization process) and insoluble systems, respectively. An open-star symbol denotes the CBDA (70); 6FDA(30)/AB-TFMB copolymer system, (■, □) conventional wholly aromatic PIs, (♦) CBDA/DABA, (▲) insoluble semi-cycloaliphatic PIs obtained using CBDA or cycloaliphatic diamines and TFMB-based PIs, (**◊**) soluble PIs obtained using H-PMDA, H″-PMDA, or HTA-HQ, (○) PIs obtained using TA-TFMB or TA-TFBP, (×) PES. Reproduced with permission from Elsevier [21].

Figure 18 shows a CTE–transparency–solubility diagram. The target area (provisionally, CTE ≤ 15 ppm·K^−1^, *T*_400_ ≥ 80%) is also established in this figure for the optical applications mentioned above. Some conventional aromatic PIs (■) have low CTE values. However, these PIs are mostly highly colored and insoluble. Optically transparent soluble PI systems that we have examined previously (◊) exhibit significantly improved heat resistance (*T*_g_) in comparison with engineering plastics such as PES (×) while maintaining good transparency. However, they do not always have low CTE values. In contrast, semi-cycloaliphatic rigid PIs such as s-BPDA/*t*-CHDA and CBDA/TFMB possess low CTE values. However, they are essentially insoluble in common solvents, therefore, stable PI solutions are not obtained at room temperature. Figure 18 also suggests that these PIs tend to lose both transparency and solubility below a CTE of ca. 20 ppm·K^−1^. On the other hand, our optimized system, the CBDA(70);6FDA(30)/AB-TFMB copolymer (open-star symbol), overcomes the difficulty in simultaneously achieving low CTE, high transparency, and solution-processability (see the plot positioned within the target area). Thus, this optimized system, which achieved an ultra-low CTE of 7.3 ppm·K^−1^, high transparency (*T*_400_ = 80.6%, YI = 2.5), a very high *T*_g_ of 329 °C, sufficient ductility (*ε*_b max_ > 30%), and good solution-processability, is a promising candidate as a coating-type plastic substrate material.

### 4.5. Attempts at Fulfilling the Target Properties without Relying on Cycloaliphatic Monomers

There has been concern that semi- and wholly cycloaliphatic PIs may be inferior to aromatic PIs in terms of long-term stability (durability). This is true concerning the chemical heat resistance, as expressed by the thermal decomposition temperatures (e.g., the 5% weight loss temperatures). However, the durability for other properties is far from being fully understood because systematic investigations have not been made concerning these issues. For example, the long-term light resistance of the optical transparency is very important for practical use [82]. From these aspects, in addition to the semi-cycloaliphatic PI systems described above, we have studied how solution-processable optically transparent PIs with low CTE values can be obtained without the help of cycloaliphatic monomers and obtained some aromatic PAI and PEsI systems.

For this purpose, we synthesized an amide-linked tetracarboxylic dianhydride (TA-TFMB) derived from TFMB with trimellitic anhydride derivative [15]. This was combined with TFMB, and a highly soluble PAI (TA-TFMB/TFMB, bottom left in Figure 7) was obtained. This PAI was compatible with the chemical imidization process, despite its very rigid chain structure. The CPN-cast PI film was almost colorless, corresponding to the suppressed YI value (3.9), even though the *T*_400_ value was not very high (43–56%) [15]. In general, when a film sample absorbs blue-violet light, which corresponds to light at a wavelength range of 420–450 nm, the film is yellow-colored (yellow is a complementary color of blue-violet light). The low yellowness of the above-mentioned PAI film is associated with the fact that the light transmittance (T %) curve rises steeply when changing the wavelength from 380 to 420 nm, indicating the absence of absorption in the range of 420–450 nm (blue-violet light). Therefore, this PAI film appears almost colorless. This film also displays an extremely low CTE (9.9 ppm·K^−1^), a very high *T*_g_ (328 °C), and sufficient film ductility (*ε*_b max_ = 27%) [15] (Table 1, sample 16). This PAI has the same chemical composition as a random-sequence PAI conveniently obtained using commercially available TFMB and trimellitic anhydride chloride (TMAC). However, the latter is obviously inferior to TA-TFMA/TFMB in all the target properties. Thus, the regularity of the chain sequence is also an important factor. The results also emphasize the importance of the molecular design of the monomers again, as demonstrated by the use of TA-TFMB.

We also synthesized an ester-linked tetracarboxylic dianhydride (TA-TFBP), which was then combined with TFMB. The obtained PEsI (TA-TFBP/TFMB, bottom left in Figure 7) was highly soluble, as was the PAI counterpart. The PEsI film prepared via chemical imidization has further improved optical transparency (*T*_400_ = 70.7%), a low CTE (Table 1, sample 17), and an extremely low water uptake (0.03%). In this case, further improvement of the CTE by copolymerization with rigid aromatic monomers seems to be unlikely. For example, the use of PMDA as a comonomer very likely causes significant deterioration of the original properties of TA-TFBP/TFMB, i.e., prominent coloration and a significant decrease in the solubility, although the CTE could be reduced. Surprisingly, copolymerization using NTDA (30 mol %) as a selected modifier enables us to further reduce the CTE (13 ppm·K^−1^) while maintaining the originally high optical transparency (Table 1, sample 18), although the resultant copolymer became insoluble in CPN (but was still highly soluble in amide solvents).

We have previously found, in the course of the development of dielectric films for flexible printed circuits, that an ester-linked aromatic tetracarboxylic dianhydride (TA-HQ) derived from HQ and TMAC tends to offer pale-colored films after thermal imidization, compared to conventional aromatic PI films [47]. In particular, the TA-HQ/TFMB system (Table 1, sample 14) leads to a film with suppressed coloration like that of the s-BPDA/TFMB film. These results motivated us to evaluate whether TA-HQ and its analogs have the potential for achieving our targets. Thus, the substituent effects were examined by varying the type and the number of substituents introduced to the HQ unit (methyl, methoxy, *tert*-butyl, amyl, and norbornane groups). Among the substituents examined, the 2,3,5-trimethyl, 2,5-di-*tert*-butyl, and 2,5-diamyl substituents (bottom right in Figure 7) are very effective in improving the solubility [21]. These PEsI systems offer highly transparent films through chemical imidization. In particular, the CPN-cast film for the system derived 2,3,5-trimethyl-substituted TA-HQ (i.e., TA-TMHQ) and TFMB (Table 1, sample 15) achieves excellent combined properties: a very low CTE (12 ppm·K^−1^), relatively high transparency (*T*_400_ = 65%), a relatively high *T*_g_ (276 °C by TMA), a very low water uptake (0.13%), and sufficient film ductility (*ε*_b max_ = 27%). A further increase in the *T*_g_ without sacrificing the other target properties is an important issue for consideration [83]. Thus, it is possible to achieve most of the target properties without using cycloaliphatic monomers through the combination of molecular design and the control of the processing conditions.

## 5. Conclusions

This paper has reviewed the research and development of new high-temperature polymeric materials suitable as plastic substrates in image display devices with a focus on our previous results, and novel solution-processable, low-CTE, colorless PIs have been proposed.

Conventional aromatic PI films are highly colored owing to the CT interactions. The 6FDA/TFMB system is an unusual case that leads to a practically non-colored PI film. However, this PI film does not have a low CTE owing to the distorted steric structure of 6FDA. The most effective strategy for completely removing the film coloration is to use cycloaliphatic monomers either in diamines or tetracarboxylic dianhydrides or both, whereby the CT interactions practically disappear. However, great attention should also be paid to the processing conditions of the polymerization, imidization, and film preparation processes (e.g., temperature, atmosphere, type of solvent, and the presence of unknown colored impurities originally contained in the monomers).

The most important factor for reducing the CTE is the main chain linearity of PIs. The in-plane chain orientation is induced by thermal imidization of the PAA cast films formed on substrates. The imidization-induced in-plane orientation behavior occurs more prominently when the systems have higher main chain linearity. However, most low-CTE PI systems with rigid structures are intensely colored.

A possible solution for simultaneously achieving high transparency and low-CTE property is the use of semi-cycloaliphatic PI systems with linear chain structures. However, when using cycloaliphatic diamines (particularly, *t*-CHDA), salt formation occurs in the initial stages of PAA polymerization, which terminates the polymerization in some cases or significantly extends the reaction period. Among PI systems using cycloaliphatic diamines, s-BPDA/*t*-CHDA is a promising system for our purpose because of its relatively good properties [*T*_400_ = ~80%, a high *T*_g_ (>300 °C), and a very low CTE (10 ppm·K^−1^)] except for its insufficient film toughness (*ε*_b max_ ~10%).

On the other hand, in the combinations of cycloaliphatic tetracarboxylic dianhydrides and aromatic diamines, high-*M*_w_ PAAs can be easily obtained without salt formation. The steric structures of cycloaliphatic tetracarboxylic dianhydrides significantly influence the polymerizability with aromatic diamines and the properties (particularly, CTE). For three H-PMDA isomers, a steric effect on the polymerizability and the properties of the resultant PI films were discussed. H′-PMDA and CBDA (particularly, CBDA) are limited cycloaliphatic tetracarboxylic dianhydrides that are suitable for reducing the CTE. For example, the CBDA/TFMB system led to a colorless PI film with a relatively low CTE (21 ppm·K^−1^). However, this PI system is insoluble in common organic solvents (i.e., the absence of solution-processability and the chemical imidization process compatibility) and led to a somewhat brittle film (*ε*_b_ < 10%).

In addition, the film preparation route had a strong effect on the properties. The films prepared via chemical imidization always showed higher transparency and lower CTE than those via the conventional two-step process. The results suggest that compatibility with the chemical imidization process holds the key to achieving our present goals. Therefore, our efforts have been devoted to dramatically improving the solubility of the CBDA-based PI systems. For this purpose, we have designed and synthesized a new diamine (AB-TFMB), which has the structural features of TFMB and DABA. The CBDA(70);6FDA(30)/AB-TFMB copolymer system overcame the difficulty in simultaneously achieving the target properties and accomplished an ultra-low CTE of 7.3 ppm·K^−1^, high transparency (*T*_400_ = 80.6%, YI = 2.5, and haze = 1.5%), a very high *T*_g_ of 329 °C, sufficient ductility (*ε*_b max_ > 30%), and good solution-processability. Therefore, this copolymer system is a promising candidate for a novel coating-type plastic substrate material.

This paper also discussed how the target properties could be achieved without the help of cycloaliphatic monomers, and the use of elaborate molecular designs yielded transparent and low-CTE aromatic PAI and PEsI systems.

## Figures and Tables

**Table 1:** Salt formation behavior in the PAA polymerization and selected properties of PI films for the systems that we have investigated so far.

No.	System ^a^	Salt formation	Chemical imidization process	Appearance of PI films	CTE ^b^ (ppm·K^−1^)	*T*_g_ (°C)	Reference
1	PMDA/TFMB	None	Non-applicable	Highly colored	–5 (T)	400	[27,46,50]
2	6FDA/TFMB	None	Applicable	Colorless	53 (C) 64 (T)	324–335	[15,27,45]
3	s-BPDA/TFMB	None	Non-applicable	Slightly colored	34 (T)	314	[45,46]
4	CBDA/*t*-CHDA	Very strong	Non-applicable	Colorless	26 (T)	ND ^c^	[17]
5	s-BPDA/*t*-CHDA	Strong	Non-applicable	Colorless	10 (T)	360	[45]
6	PMDA/*t*-CHDA	Very strong	Non-applicable	Colorless	10 (T)	ND ^c^	[17]
7	CBDA/TFMB	None	Non-applicable	Colorless	21 (T)	356	[36,45]
8	DM-CBDA/TFMB	None	Non-applicable	Colorless	28 (T)	341	[36]
9	HTA-44BP/*o*-TOL	None	Applicable	Colorless	63 (C)	295	[35]
10	H′-PMDA/TFMB	None	Applicable	Colorless	30–46 (C)	357	[37]
11	H′-PMDA/AB-TFMB	None	Applicable	Colorless	25 (C)	340	[51]
12.	CBDA(70); 6FDA(30)/AB-TFMB	None	Applicable	Colorless	7.3 (C)	329	[51]
13	CBDA(80); 6FDA(20)/AB-TFMB	None	Applicable	Colorless	4.2 (C)	335	[51]
14	TA-HQ/TFMB	None	Non-applicable	Slightly colored	23 (T)	370	[21,48]
15	TA-TMHQ/TFMB	None	Applicable	Almost colorless	12 (C)	276	[21]
16	TA-TFMB/TFMB	None	Applicable	Almost colorless	10 (C)	328	[15]
17	TA-TFBP/TFMB	None	Applicable	Almost colorless	20 (C)	261	[15]
18	TA-TFBP(70); NTDA(30)/TFMB	None	Applicable	Almost colorless	13 (C)	277	[15]

^a^ The structures of the abbreviated systems are indicated in Figure 5, Figure 6 and Figure 7 and the text. ^b^ Data for the PI films via thermal imidization (T) and chemical imidization (C). ^c^ Not detected by dynamic mechanical analysis.
